# The Expression Level of SOX Family Transcription Factors’ mRNA as a Diagnostic Marker for Osteoarthritis

**DOI:** 10.3390/jcm14041176

**Published:** 2025-02-11

**Authors:** Kamila Baran, Ewa Brzeziańska-Lasota, Jakub Kryczka, Joanna Boncela, Aleksandra Czechowska, Karolina Kopacz, Gianluca Padula, Krzysztof Nowak, Marcin Domżalski

**Affiliations:** 1 Department of Biomedicine and Genetics, Chair of Biology and Medical Microbiology, Medical University of Lodz, 92-215 Lodz, Poland; ewa.brzezianska@umed.lodz.pl; 2 Institute of Medical Biology, Polish Academy of Sciences, 93-232 Lodz, Poland; jkryczka@cbm.pan.pl (J.K.); jboncela@cbm.pan.pl (J.B.); 3 Academic Laboratory of Movement and Human Physical Performance, Medical University of Lodz, 90-001 Lodz, Poland; aleksandra.czechowska@umed.lodz.pl (A.C.); karolina.kopacz@umed.lodz.pl (K.K.); gianluca.padula@umed.lodz.pl (G.P.); 4 Department of Orthopedics and Traumatology, University Clinical Hospital No. 2 of the Medical University of Lodz, 90-549 Lodz, Poland; krzysztof.nowak@umed.lodz.pl (K.N.); marcin.domzalski@umed.lodz.pl (M.D.)

**Keywords:** osteoarthritis, prognosis biomarker, real-time polymerase chain reaction, SOX family transcription factors

## Abstract

**Background/Objectives:** *Osteoarthritis* (*OA*) is the most common degenerative and chronic joint disease and is a leading cause of pain and disability in adults worldwide. The SRY-related HMG box (SOX) family transcription factors (TFs) play a crucial role during the pathogenesis of OA; however, their exact mechanisms remain unexplored. The aim of our study was to conduct a bioinformatics analysis of the common interactions of SOX-5, SOX-9, and SOX-11 with other proteins, as well as their role in OA pathogenesis. **Methods:** *SOX5*, *SOX9*, and *SOX11* mRNA expression levels in articular cartilage with subchondral bone and synovium from knee OA patients were assessed using the qPCR method. The study group consisted of thirty-one patients (n = 31). Total RNA was isolated from the articular cartilage with subchondral bone and synovium from the affected and unaffected area of the knee joint. **Results:** Our results revealed a regulatory network between SOX-5, SOX-9, and SOX-11, and various proteins involved in the pathogenesis of knee OA and their collective interactions, which are involved in the regulation of cartilage extracellular matrix (ECM) organization, response to stimulus, regulation of gene expression, inflammatory response, cartilage condensation, and ossification in chondrocytes. Higher expression levels of *SOX5*, *SOX9*, and *SOX11* mRNA were noted in OA-affected articular cartilage with subchondral bone compared to control tissue (*p* = 0.00015, *p* = 0.0024 and *p* > 0.05, respectively, Mann–Whitney U-test). All studied genes demonstrated elevated mRNA expression levels in the articular cartilage with subchondral bone from stage 4 patients than those with stage 3 (*p* > 0.05; Mann–Whitney U-test). Lower *SOX5*, *SOX9*, and *SOX11* mRNA expression levels were found in OA-affected synovium compared to the control tissue (*p* = 0.0003, *p* > 0.05 and *p* = 0.0007, respectively, Mann–Whitney U-test). Decreased *SOX9* mRNA expression levels in synovium were noted in patients with stage 4 disease than those with stage 3; however, *SOX5* and *SOX11* mRNA expression levels were higher in patients with stage 4 (*p* > 0.05; Mann–Whitney U-test). **Conclusions:** The results of our research show that the studied SOX TFs play a role in the development of OA, contributing to the formation of pathological changes not only in the articular cartilage, but also in the synovial membrane. The changes in the *SOX5*, *SOX9*, and *SOX11* mRNA expression levels in the articular cartilage with subchondral bone and synovium may serve as potential molecular diagnostic biomarkers for detecting OA and could indicate the progression of this disease; however, our observations require further investigation.

## 1. Introduction

Osteoarthritis (OA) is a widespread progressive and irreversible degenerative joint disease and a leading cause of functional disability. Its prevalence has increased by 113.25% worldwide over the past three decades, from 247.51 million in 1990 to 527.81 million in 2019 [1]. OA can damage any joint, but almost four-fifths of the burden of this disease is carried by the knees [2]. Among the many risk factors, knee OA is believed to be primarily affected by knee joint injury history, obesity, repetitive loading, hormonal factors (also associated with postmenopausal changes), bone density, muscle weakness, malalignment, and genetic predisposition [3]. Patients suffering from knee OA most often experience symptoms such as stiffness, swelling, limited range of motion of the knee joint and its deformity, as well as chronic, long-term pain [4].

Therapies for knee OA include a reduction in modifiable risk factors by weight loss, bracing, physical modalities, and pharmacologic treatment in the form of oral administration of acetaminophen or nonsteroidal anti-inflammatory drugs (NSAIDs), as well as intra-articular injection of corticosteroids [5]. All of these treatments may relieve symptoms and slow the progression of OA but cannot repair damaged articular cartilage. Total knee arthroplasty (TKA) is typically used as a last resort for advanced knee arthritis when non-operative treatments have failed [6]. Early detection of the disease is crucial because patients with early-stage OA of the knee respond better to treatment than patients with more severe forms, which may lead to make better treatment outcomes and inhibit progression [7]. Furthermore, a better understanding of the molecular mechanism of OA development is crucial for therapeutic target design [8,9].

One of the key pathological processes in OA is an imbalance between anabolic and catabolic processes in chondrocytes, chondrocyte hypertrophy, chondrocyte senescence, and their autophagy, which depend on intra- and extracellular signals received by the cell [10,11,12]. Transcription factors, including the SRY-related HMG box (SOX) family of transcription factors (TFs), play a central role in chondrocyte function and phenotype and are responsible for the normal development, maintenance, and degeneration of articular cartilage [12]. They harbor a high-mobility-group (HMG)-box DNA-binding domain that is at least 50% identical in sequence to that of the sex-determining region Y protein (SRY), hence their name [13]. SOX proteins are classified into ten groups (A-J) according to their structure; they are encoded by 30 SOX genes [14,15].

SOX family TFs bind to a specific sequence [(A/T)(A/T)CAA(A/T)G motifs] in the minor groove of the DNA helix, which introduce strong bends into the DNA [16]. SOX TFs play regulatory roles through activating or repressing gene transcription; this enables the SOX proteins to regulate different actions in the same cell type and to drive the developmental processes in a particular tissue [17,18].

The transcription factor SOX-9 is essential for the organogenesis and development of many human tissue, organs, and systems including the bone and skeletal system which participates in endochondral ossification [19]. During chondrogenesis, SOX-9 is expressed in all chondroprogenitors and also in all differentiated chondrocytes [11]; it is responsible for stimulating mesenchymal cell condensation, differentiating prechondrogenic precursor cells into chondrocytes, driving chondrocyte proliferation and inhibiting chondrocyte senescence [11,12,20]. Experimental ex vivo and in vitro data indicate that SOX-9 directly modulates the expression of chondrocyte-specific genes, as well as those coding for cartilage extracellular matrix (ECM) modifying enzymes [21,22]. Furthermore, SOX-9 controls the conversion of proliferating chondrocytes into hypertrophic chondrocytes, contributing to endochondral ossification and cartilage vascularization [23,24]. Two other members of the SOX family TFs, SOX-5 and SOX-6, cooperate with SOX-9 in chondrocyte differentiation and proliferation, as well as activation of *COL2A1* and *ACAN* transcription, contributing to the synthesis of collagen type II alpha 1 chain and aggrecan—the major components of the cartilage matrix [11,25]. A recent study by Xu et al. indicated that SOX-11 may also be involved in the development of OA through its effect on chondrocyte apoptosis [26]. An increased expression level of SOX-11 enhances the production of caspase-3, which cleaves many key cellular proteins, leading to cell death [26,27].

Our bioinformatics analysis shows that SOX-5, SOX-9, and SOX-11 engage in a number of interactions with other proteins involved in the pathogenesis of knee OA. The aim of the study was to evaluate changes in the mRNA expression level of three SOX family TF member genes, *viz. SOX5*, *SOX9*, and *SOX11*, in articular cartilage with subchondral bone and synovium from knee OA patients. The expression level of the studied genes was also compared in the affected and unaffected area of the knee joint to identify a potential molecular biomarker for detecting OA and the degree of disease progression. Additionally, the expression profile of *SOX5*, *SOX9*, and *SOX11* in articular cartilage with subchondral bone and synovium of OA patients was analyzed in relation to sex, age, weight, and body mass index (BMI). The relationship between the change in molecular factors and the progression of the disease may be an important diagnostic factor, enabling faster diagnosis of the disease and implementation of targeted treatment in patients with knee OA.

## 2. Materials and Methods

### 2.1. Clinical Characteristics of OA Patients

The study cohort included 31 patients (n = 31), including 21 women and 10 men aged 50 to 80 (mean age 68 ± 7 years), with height 1.46 to 1.83 m (mean 1.64 m ± 0.08 m) and weight 66 to 112 kg (mean 88 kg ± 12.12 kg), with a diagnosis of primary knee OA with predominantly medial compartment involvement. All patients underwent total knee arthroplasty at the Department of Orthopedics and Traumatology of the Medical University of Lodz, Lodz, Poland, between 2019 and 2022. The indication for surgery was an end-stage knee OA of stage 3 or 4 according to the Kellgren–Lawrence classification with clinical symptoms of OA [28]. The inclusion and exclusion criteria for the study are presented in Table 1.

The clinical characteristics of the study group, i.e., patients with diagnosed primary knee OA, are shown in Table 2.

### 2.2. Biological Material

During knee arthroplasty, fragments of articular cartilage with a layer of subchondral bone and fragments of the synovium (in a volume up to 1 cm^3^) were collected from all qualified patients (n = 31). The biological material was collected from the affected area (medial compartment of the knee joint) and the unaffected area (lateral compartment of the knee joint), which served as a control in the study. The collected tissue fragments were stored in Falcon tubes containing RNA stabilizing buffer RNAlater^®^ Solution (Ambio, Applied Biosystems, Austin, TX, USA) at 4 °C during transport from the operating theater to the molecular laboratory. After removing the biological material from the stabilizing buffer, it was fragmented and then placed at −80 °C for 24 h.

### 2.3. RNA Isolation and Qualitative/Quantitative RNA Evaluation

Before proceeding with the isolation process, fragments of articular cartilage together with the subchondral bone layer were crushed using a laboratory mortar. Subsequently, the prepared fragments of articular cartilage, together with the subchondral bone layer and fragments of synovium, were homogenized using an IKA homogenizer (IKA^®^ Werke GmbH & Co. KG, Staufen, Germany). Total RNA from tissue homogenates was isolated with the use of the mirVana™ miRNA Isolation Kit (Life Technologies, Carlsbad, CA, USA) according to the manufacturer’s protocol. Qualitative and quantitative assessment of the RNA was conducted spectrophotometrically (260/280 nm) with an Eppendorf BioPhotometerTM Plus apparatus (Eppendorf, Hamburg, Germany).

### 2.4. Relative Expression Level (RQ) of Studied Genes

Reverse transcription (RT) for genes was performed with a High-Capacity cDNA Reverse Transcription Kit (Applied Biosystems, Foster City, CA USA) in accordance with the manufacturer’s protocol. The RT reaction was performed in a Personal Thermocycler (Eppendorf, Hamburg, Germany).

The relative expression level (RQ) of the genes was determined with a 7900HT Fast Real-Time PCR System (Applied Biosystems, Carlsbad, CA, USA) using TaqMan assay: *SOX5* (Hs00374709_m1), *SOX9* (Hs00165814_m1), *SOX11* (Hs00846583_s1), as well as *HPRT1* (Hs02800695_m1), which was used as an endogenous control for genes.

The relative expression levels of the genes in the fragments of the articular cartilage with the subchondral bone layer, and in the fragments of synovium, were calculated by the delta–delta CT method (TaqMan Relative Quantification Assay software v3.01, Applied Biosystems). Reference total RNA from human chondrocytes—HC-a tRNA (ScienCell Research Laboratories, Carlsbad, CA, USA) and synovium (OriGene Technologies, Rockville, MD, USA) were used as calibrators: their expressions were regarded as RQ = 1.

### 2.5. Statistical Analysis

The median values of the expression levels (RQ value) of the studied genes are presented. For all statistical analyses, the level of significance was assumed to be *p* < 0.05. The normality of data were tested using the Shapiro–Wilk test. Non-parametric tests were used for the analysis: two-group comparisons used the Mann–Whitney U-test, and for multiple group comparisons, the Kruskal–Wallis test was used. Statistical analysis was performed using Statistica 13 software (StatSoft, Cracow, Poland).

### 2.6. Microarray Data Processing and Analysis

Transcriptomic data associated with patient chondrocyte and synovium tissue deposited in GSE179716 and GSE206848 (accessed on 2 November 2023) were downloaded from the Gene Expression Omnibus (GEO) database (http://www.ncbi.nlm.nih.gov/geo/, accessed on 2 November 2023) and analyzed using the GEO2R online analytical tool (# Version info: R 4.2.2, Biobase 2.58.0, GEOquery 2.66.0, limma 3.54.0), as described previously [30,31].

### 2.7. Construction of Protein–Protein Interaction (PPI) Network

A PPI network of differently expressed genes (DEGs) was created using the STRING version 12.0 online software (https://string-db.org/, accessed on 2 November 2023) and visualized by the open-source software platform for visualizing complex networks Cytoscape version 3.9.1 (https://cytoscape.org/, accessed on 2 November 2023). A Gene Ontology Biological Processes (GO BP) analysis was performed using the Gene Ontology (GO) database (https://www.geneontology.org/, accessed on 2 November 2023). In addition, a KEGG (Kyoto Encyclopedia of Genes and Genomes) pathway analysis of the obtained network was performed using the STRING version 11.0 online software and the DAVID online tool (https://david.ncifcrf.gov/, accessed on 2 November 2023) and the KEGG PATHWAY database (https://www.kegg.jp/kegg/pathway.html, accessed on 2 November 2023), as described previously [31].

## 3. Results

### 3.1. Protein–Protein Interaction in Chndrocytes and Synovium

The transcriptomic data from the GEO database were used to identify potential proteins that interact with the SOX family TFs and to analyze their involvement in pathogenesis, progression, and course of knee OA. The GSE179716 dataset containing mRNA expression levels of early and late OA-affected chondrocytes was downloaded and analyzed; following this, the data from GSE206848 containing mRNA expression of OA-affected and normal (unaffected) synovium cells were analyzed. The GSE179716 is composed of n = 3 early and n = 3 late OA (University of Pittsburgh), whereas the GSE206848 is composed of n = 7 normal synovium samples and n = 9 late OA synovium samples (NYU Grossman School of Medicine).

**Figure 1:**
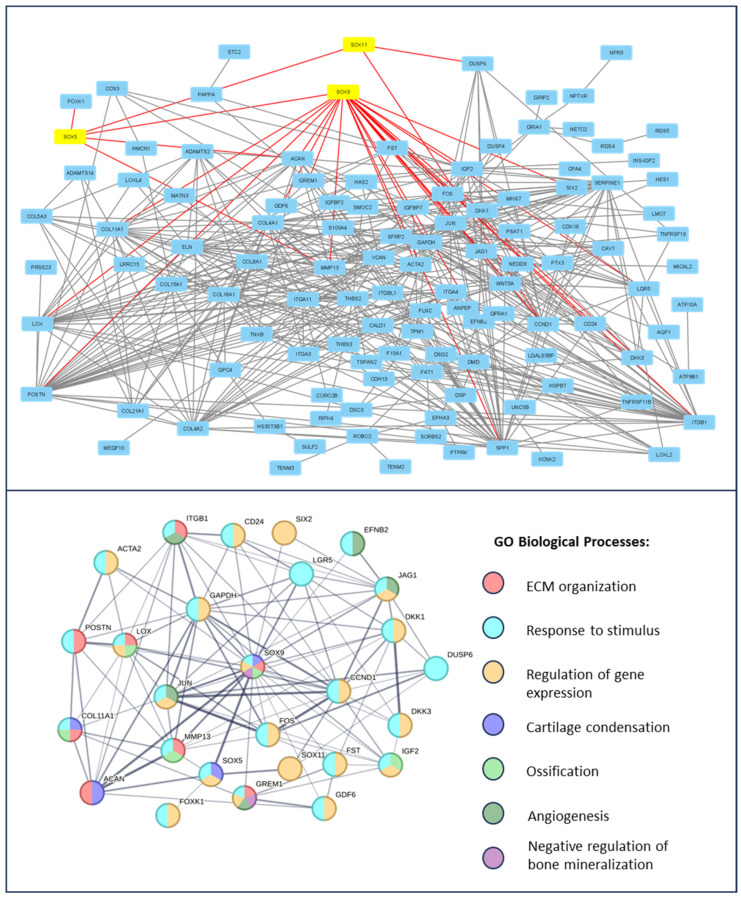
Enrichment analysis of SOX transcription factors in the course of knee OA in chondrocytes. A PPI network of top up-regulated genes, related to OA progression, is presented on the upper panel. Studied SOX family TFs are highlighted in yellow and the direct protein interactions are indicated by red lines. The PPI network of the direct SOX family TFs interactions and, thus, the resulting network’s direct enrichment of Gene Ontology Biological Processes, is presented on the lower panel. Data were obtained from the GSE179716 datasets and analyzed using the STRING version 11.0 online software and Cytoscape platform.

**Figure 2:**
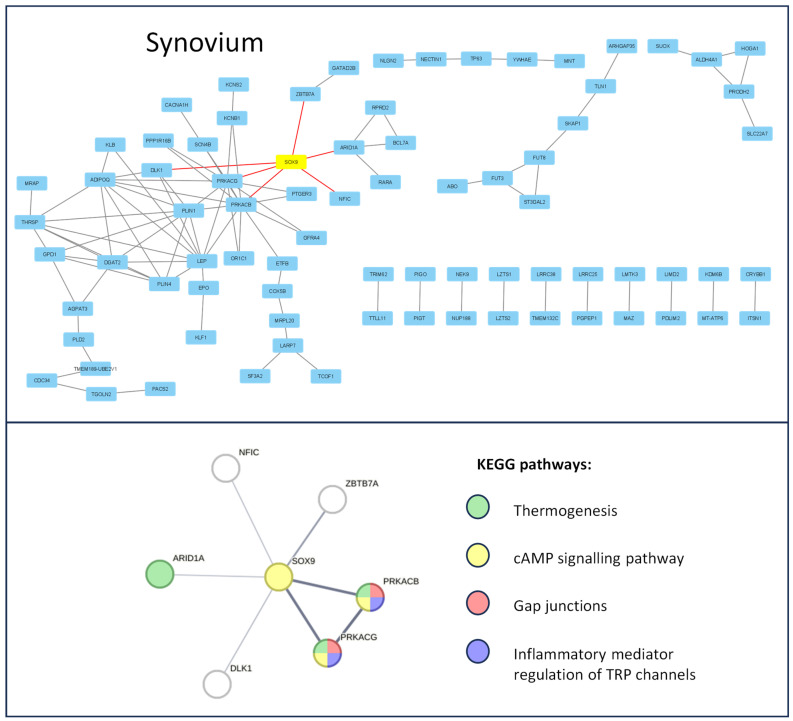
Enrichment analysis of the SOX-9 transcription factor during the course of knee OA in synoviocytes. The PPI network of top up-regulated genes, related to OA progression, is presented in the upper panel. SOX-9 is highlighted in yellow and its direct protein interactions are indicated by red lines. The PPI network of the direct SOX-9 interactions thus created the network’s direct enrichment of the KEEG pathways, which are presented on the lower panel. Data obtained from the GSE206848 dataset were analyzed using the STRING version 11.0 online software and Cytoscape platform.

First, differentially expressed genes (DEGs) for all datasets were selected with *p*. adj value < 0.05 using the GEO2R online analytical tool. The data structure is presented in (Supplementary Materials, Figure S1). A protein–protein interaction (PPI) network was then constructed for 200 significantly top-upregulated DEGs in late OA-affected chondrocytes (Figure 1) and synovium (Figure 2); this was performed using STRING version 11.0 online software, and the data were visualized using Cytoscape platform (excluding nods without any protein–protein interaction). SOX transcription factor family members were highlighted as yellow and their direct connections to significant DEGs were highlighted as red.

The Gene Ontology Biological Processes analysis of the SOX-related interaction web revealed the following major dysregulated pathways for chondrocytes affected by OA: ECM organization (GO:0030198), response to stimulus (GO:0050896), regulation of gene expression (GO:0010468), angiogenesis (GO:0001525), negative regulation of bone mineralization (GO:0030502), cartilage condensation (GO:0001502), and ossification (GO:0001503). In contrast, in the OA-affected synovium, no GO BP enrichment was observed. Therefore, a KEGG analysis was performed. The results indicate that the following were dysregulated: thermogenesis (hsa04714), cAMP signaling pathway (hsa04024), gap junctions (hsa04540), and inflammatory mediator regulation of TRP channels (hsa04750). Proteins involved in the regulation of the mentioned pathways, highlighted according to the Gene Ontology Biological Processes, and KEGG pathway color are presented in Figure 2.

### 3.2. Relative Expression Level (RQ) of SOX5, SOX9, and SOX11 mRNA in Articular Cartilage with Subchondral Bone in OA Patients vs. Control Tissue

**Figure 3:**
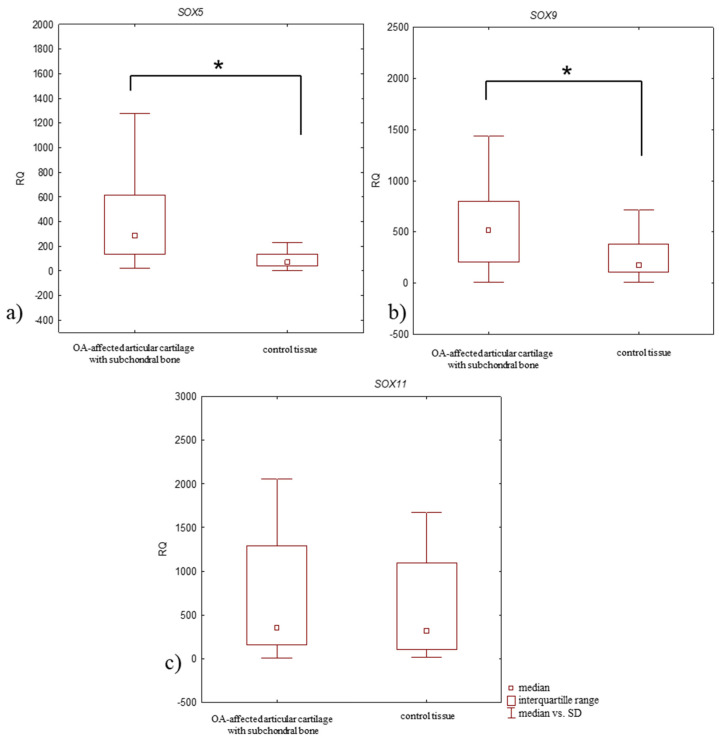
Box plot presenting differences in median RQ values for (**a**) *SOX5*, (**b**) *SOX9*, (**c**) *SOX11* in OA-affected articular cartilage with subchondral bone vs. control tissue. * *p* < 0.05.

Upregulated (RQ > 1) SOX5, SOX9, and SOX11 mRNA expression levels were observed in all OA-affected articular cartilage with subchondral bone and in all control tissue. *SOX5* mRNA expression level was significantly higher in OA-affected articular cartilage with subchondral bone than in control tissue (median RQ: 287.935 and 69.395, *p* = 0.00015; Mann–Whitney U-test) (Figure 3a, Table 3), as were the *SOX9* mRNA level (median RQ: 522.929 and 178.271, respectively; *p* = 0.0024, Mann–Whitney U-test) (Figure 3b, Table 3). *SOX11* mRNA expression level was also elevated in OA-affected articular cartilage with subchondral bone compared to control tissue (median RQ: 354.660 and 317.992); however, the difference in expression level was statistically insignificant (*p* > 0.05, Mann–Whitney U-test) (Figure 3c, Table 3).

### 3.3. Relative Expression Level (RQ) of Studied Genes in Articular Cartilage with Subchondral Bone of OA Patients According to Clinical Characteristics of Patients

Our evaluation of studied gene expression levels in OA-affected articular cartilage with subchondral bone according to clinical characteristics of patients and advancement of the disease revealed differences in SOXs expression level between study groups.

In relation to gender, a higher expression level of *SOX5* mRNA in OA-affected articular cartilage with subchondral bone was noted among more men than women (median RQ: 339.090 and 220.128); whereas, the samples from women demonstrated higher *SOX9* and *SOX11* mRNA expression levels (Table 3). Even so, the observed differences in expression level between all studied genes were statistically insignificant (*p* > 0.05, Mann–Whitney U-test).

Regarding age, the expression levels of *SOX5* and *SOX9* mRNA in OA-affected articular cartilage with subchondral bone were statistically significantly lower in patients aged >65 than those ≤65 (*p* = 0.04 and *p* = 0.017, for *SOX5* and *SOX9*, respectively; Mann–Whitney U-test) (Table 3). A lower *SOX11* mRNA expression level was also noted in the patients aged >65 compared to ≤65, but the differences were statistically insignificant (*p* > 0.05, Mann–Whitney U-test).

Relative to the BMI, *SOX5* mRNA expression level was lowest in samples from overweight patients and highest in those with class II + III obesity (Table 3). Similarly, the *SOX9* mRNA expression level was highest in patients with class II + III obesity; however, it lowest in patients with class I obesity. Finally, *SOX11* mRNA expression level was the highest in overweight patients, but lowest in those with class I obesity. The differences between the expression level of all studied genes were statistically insignificant (*p* > 0.05, Mann–Whitney U-test).

**Figure 4:**
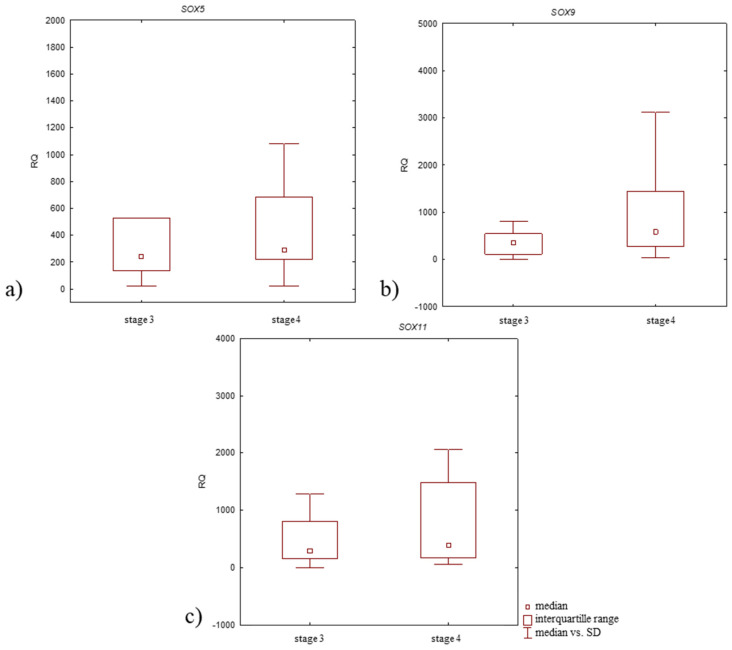
Box plot presenting differences in median RQ values for (**a**) *SOX5*, (**b**) *SOX9*, (**c**) *SOX11* in OA-affected articular cartilage with subchondral bone according to the stage of disease development (stage 3 and 4 according to Kellgren–Lawrence classification).

Focusing on the stage of OA development, all studied genes demonstrated elevated mRNA expression levels in samples from patients with stage 4 of disease compared to those with stage 3 (Figure 4, Table 3); however, the differences in the expression level of all tested genes between study groups were statistically insignificant (*p* > 0.05, Mann–Whitney U-test).

### 3.4. Relative Expression Level (RQ) of the SOX5, SOX9, and SOX11 mRNA in Synovium of OA Patients vs. Control Tissue

**Figure 5:**
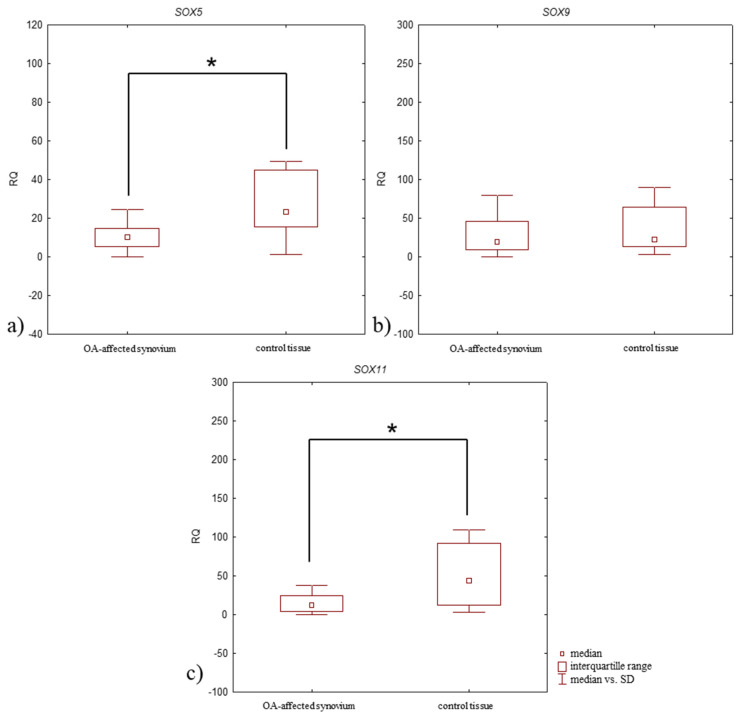
Box plot presenting differences in median RQ values for (**a**) SOX5, (**b**) SOX9, (**c**) SOX11 in OA-affected synovium vs. control tissue. * *p* < 0.05.

Upregulated (RQ > 1) SOX5 and SOX9 mRNA expression levels were found in 97% of OA-affected synovium, while an upregulated expression level of SOX11 mRNA was found in 87%. In the control tissue, the upregulated expression level of all studied genes were in 100% of synovium. The *SOX5* mRNA expression level was significantly lower in OA-affected synovium compared to control tissue (median RQ: 10.193 and 23.297, respectively, *p* = 0.0003, Mann–Whitney U-test) (Figure 5a, Table 4). *SOX11* mRNA expression level was also significantly decreased in OA-affected synovium compared to the control tissue (median RQ: 12.436 and 44.471, respectively, *p* = 0.0007, Mann–Whitney U-test) (Figure 5c, Table 4). *SOX9* mRNA expression level was also lower in OA-affected synovium than the control tissue (median RQ: 19.722 and 22.874, respectively), although this difference was statistically insignificant (*p* > 0.05, Mann–Whitney U-test) (Figure 5b, Table 4).

### 3.5. Relative Expression Level (RQ) of Studied Genes in Synovium of OA Patients According to the Clinical Characteristics of the Patients and Pathological Tissue Examination

The samples from women demonstrated higher *SOX5* and *SOX9* mRNA expression levels in OA-affected synovium than those from men (Table 4), but *SOX11* mRNA expression levels were higher in men. All differences between the expressions level of all studied genes were statistically insignificant (*p* > 0.05, Mann–Whitney U-test).

Higher *SOX5*, *SOX9*, and *SOX11* mRNA expression levels were observed in OA-affected synovium in patients aged ≤65 compared to those aged >65 (Table 4). The differences in studied gene expression levels between ages were statistically insignificant for all genes (*p* > 0.05, Mann–Whitney U-test).

The lowest *SOX5* and *SOX9* mRNA expression levels in OA-affected synovium were noted in overweight patients, and the highest in patients with class II + III obesity (Table 4). The lowest *SOX11* mRNA expression level was found in overweight patients, and the highest in those with class I obesity. The differences in expression level between weight classes were statistically insignificant for all genes (*p* > 0.05, Mann–Whitney U-test).

**Figure 6:**
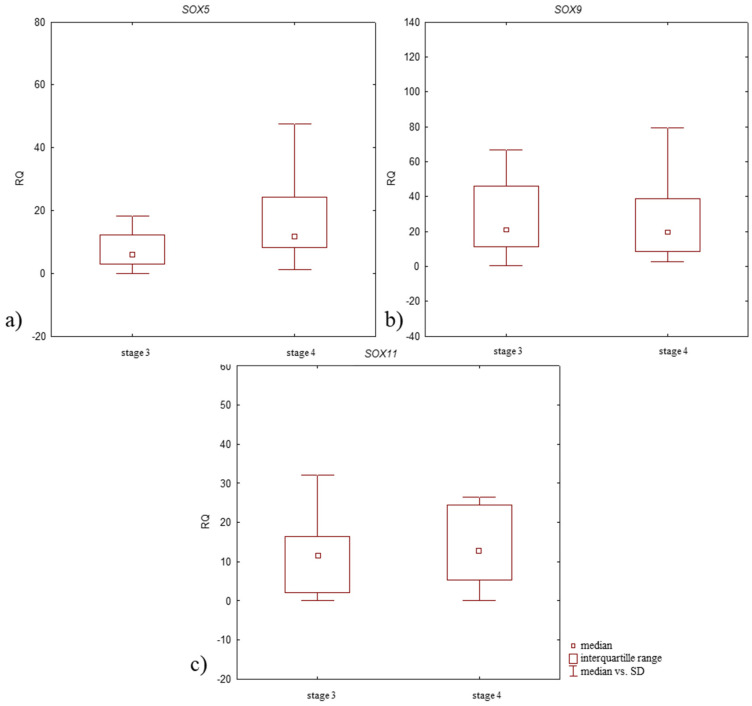
Box plot presenting differences in median RQ values for (**a**) SOX5, (**b**) SOX9, (**c**) SOX11 in OA-affected synovium according to the stage of disease development (stage 3 and 4 according to Kellgren–Lawrence classification).

Higher *SOX5* and *SOX11* mRNA expression levels were noted in patients with stage 4 disease compared to those with stage 3. *SOX9* mRNA expression level was lower in patients with stage 4 disease (Figure 6, Table 4). All differences in expression level between patient groups were insignificant (*p* > 0.05, Mann–Whitney U-test).

## 4. Discussion

OA is a widely prevalent chronic joint disease characterized by articular cartilage degeneration, subchondral bone remodeling, osteophyte formation, and synovial inflammation [32]. It is well known that knee OA is a multiple factors disease, which includes unmodifiable factors, such as age, sex, and modifiable factors, i.a. obesity, knee injury. The meta-analysis by Ciu et al. showed that the risk of knee OA increases with age and reaches a peak at age 70–79 [33]. Moreover, it was revealed this disease is approximately 1.7 times more common in women than in men, which may be caused by women having thinner articular cartilage and lower knee cartilage volume than men [34,35]. Women present lower congruity index values and higher normalized contact areas than men, as well as having a greater tendency to have uneven mechanical load, joint instability, and varus, which may support development of knee OA [34,36]. Obesity, a well-known global epidemic, is the greatest modifiable risk factor for OA, as revealed the study by Coggon et al., individuals with a BMI >30 kg/m2 were 6.8 times more likely to develop knee OA than normal-weight controls [37,38]. Recent studies have also implicated obesity in the pathogenesis of knee OA, not only through increased mechanical loading but also through the systemic effects of obesity-induced inflammation [37]. An increased incidence of knee OA has also been observed in workers with physically demanding occupations exposed to several biomechanical stressors, such as knee bending, kneeling or crouching, standing for long hours (≥2 h/day), walking ≥ 3 km/day, regularly climbing stairs, lifting weights (≥10 kg), jumping, and vibrations [39]. Participation in sports may increase the risk of knee OA, especially in football players, weightlifters, long-distance runners, and wrestlers [40]. Interestingly, the incidence of knee OA was negatively associated with the level of education, which may be due to the fact that people with lower education were more likely to engage in vigorous physical activity or had limited access to knowledge about the prevention of knee OA. To date, there is no therapy that effectively stops the structural degradation of cartilage and bone or can effectively reverse existing structural defects. The limited treatment options for OA ultimately leads to disability, which has a considerable negative effect on quality of life [41,42].

The diagnosis of OA is mainly made clinically, often confirmed by radiography, however biochemical markers are not used in primary care [43]. Research is ongoing to identify biochemical and immunological biomarkers that can monitor disease progression and predict response to pharmacological, non-pharmacological, and biological treatments [43,44]. The most commonly studied biomarkers of OA include cartilage matrix breakdown products, such as collagen type II C-telopeptide (CTX-II) and cartilage oligomeric matrix protein (COMP), inflammatory biomarkers, including C-reactive protein (CRP), interleukin-6 (IL-6), and tumor necrosis factor-alpha (TNF-α), as well as metabolic biomarkers (adiponectin, leptin, and insulin), and biomarkers of bone remodeling, such as type I collagen cross-linked N-telopeptide (NTX) and bone-specific alkaline phosphatase (BSAP) [43]. To date, none of the biochemical markers studied have been found to be sufficiently discriminating enough to aid in the diagnosis and prognosis of OA in individuals or limited numbers of patients, nor perform consistently enough to be used as an outcome in clinical trials [43,44]. Therefore, it is so important to conduct further study on the main molecular mechanisms causing the initiation and progression of OA. Recently, Emad and Abdullah identified SOX4 among SOXC TFs as a novel therapeutic target and early diagnostic marker in the pathogenesis of OA [45]. They presented the results of studies indicating higher expression levels of SOX4 in chondrocyte models of OA and in OA patients, which may be caused by pro-inflammatory factors such as IL-1β or advanced glycation end products (AGEs), stimulating chondrocytes to produce SOX4 in a dose-dependent manner. Furthermore, they emphasized that the role of SOX11 in OA is still unclear; however, due to the significant dysregulation of its expression level during disease progression, more mechanistic details are still needed. Based on the currently available data, we selected SOX TFs as promising targets for OA research for biomarker discovery and focused on understanding the exact role of three of them: SOX5, SOX9, and SOX11.

Articular cartilage is the connective tissue that covers the ends of the bones in the joints. It is crucial for allowing smooth and friction-free movement, and acts as a shock absorber during movement. However, it is one of the most difficult tissues to regenerate due to its poor ability to self-repair, resulting in tissue loss that is generally permanent and progressive [46]. The function of articular cartilage is supported by the activity of chondrocytes, which synthesize and directly interact with their surrounding ECM, forming its unique structure. During the development of OA, the metabolic activity of chondrocytes shifts to produce more catabolic factors involved in cartilage degradation, thus changing the molecular composition and organization of the ECM in the articular cartilage [47]. Chondrocyte function and phenotype are attributed to the intra- and extracellular signals they receive that alter the expression of various genes, including by modulating the activity of TFs [12,48]. Several mechanisms are known to modulate the function of TFs and thereby coordinate specific gene expression, including interactions with other TFs or cofactors [49,50]. Errors in the mechanisms of the TF network or mutations in their DNA binding sites may contribute to development of various diseases, including OA, demonstrating their important role in cartilage homeostasis [48,51].

Many SOX family TFs are recognized as master regulators of chondrogenesis [52,53,54,55,56,57,58]. They are known to regulate various phases of chondrocyte development such as early mesenchymal condensation and proliferation, modulation of chondrocyte hypertrophy, chondrocyte senescence, and autophagy [12]. Hence, selective modulation of the SOX family TFs’ expression or activity may be a promising strategy for encouraging cartilage repair. Therefore, in this study, the functional networks of selected SOX family TFs with other proteins involved in the pathogenesis of knee OA and their expression at the mRNA level were investigated.

Our KEGG pathway analysis revealed that SOX-9 is involved in the regulation of various processes, including cartilage condensation, as confirmed previously [11,59,60]. SOX-9 is expressed in all cartilage primordia during embryogenesis and has an essential function in the subsequent differentiation of prechondrogenic precursor cells into chondrocytes [59,60]. SOX-9 haploinsufficiency in humans results in campomelic dysplasia, a lethal skeletal malformation syndrome and XY sex reversal [59,61]. A study by Akiyama et al. demonstrated that SOX-9 is also required during subsequent steps of the chondrocyte differentiation pathway [11]. Inactivation of *Sox9* (using the Cre/loxP recombination system) in the limb buds of mouse embryos before mesenchymal condensation resulted in a complete absence of both cartilage and bone; however, after this process, mesenchymal cells with the inactivated *Sox9* gene did not undergo differentiation into chondrocytes and the embryos exhibited severe generalized chondrodysplasia.

Our bioinformatics analysis also confirmed that SOX-5 has a functional involvement in cartilage condensation. However, an in vivo study by Smiths et al. revealed that progenitor cell condensation occurred properly in mice with double-knockout of the *SOX5* gene [56]. The exact role of SOX-5 is difficult to indicate because it cooperates with SOX-9 to drive chondrogenesis [62]. SOX-9 induces transcription of SOX-5, in turn, SOX-5, along with SOX6, enhances the effects of SOX-9 [63]. These proteins act as architectural organizers of transcription complexes.

Our functional analysis shows that SOX-9 was involved in regulation of ECM organization. Neefjes et al. demonstrated that chondrocytes increased the production of ECM proteins through the enhanced activation of SOX-9 in early OA [12]. It was revealed the sirtuin 1 (SIRT1) acetylate SOX-9 results in the recruitment of histone acetyltransferaze (HAT) cofactors such as PGC-1, p300, and GCN5, which are proteins that hyperacetylate surrounding histones and increase *COL2A1* mRNA expression levels [64]. Further analysis by Kypriotou et al. found that SOX-9 plays a dual role in regulating *COL2A1* transcription, depending on its expression level [65]. A low increase in the level of *SOX9* expression was able to enhance transcription of the *COL2A1* gene in chondrocytes; however, a high increase in its level of expression induced inhibition of *COL2A1* gene expression [65]. Other studies revealed SOX-9 also binds to the promoter of chondrocyte marker genes such as *COL2A1*, *COL9A2*, *COL11A2*, and *ACAN*, and regulates their expression level [66,67,68]. SOX-5 and SOX-6 were also revealed to cooperate with SOX-9 in regulating *COL2A1* and *ACAN* expression [66,69,70]. Furthermore, SOX-9 regulates the expression level of ECM-degrading enzymes and is involved in the modulation of cartilage destruction in OA [71]. Zhang et al. showed the expression level of genes encoding different members of a disintegrin and metalloproteinase with thrombospondin motifs (ADAMTS) family, *viz. ADAMTS*4, *ADAMTS*5, *ADAMTS*7, and *ADAMTS*12, had negatively correlation with *SOX9* mRNA expression level in chondrocytes isolated from cartilage samples of OA patients. Their comprehensive study confirmed the regulatory function of SOX-9 on chondrocyte catabolic activity.

Moreover, our functional analysis also revealed that SOX-9 plays a vital role in the regulation of ossification in chondrocytes. Dy et al. found that SOX-9 delayed prehypertrophy of chondrocytes and prevented their osteoblastic differentiation by lowering the expression level of RUNX family transcription factor 2 (RUNX2) and inhibiting β-catenin signaling [72]. However, other studies revealed that SOX-9 promoted chondrocyte hypertrophy via interactions with myocyte enhancer factor 2C (MEF2C), Fos-related antigen 2 (FOSL2), and transcription factor JUN, and together, they directly activate the *COL10A1* promoter, which is a master regulator of this process and a specific marker of hypertrophic chondrocytes [72,73]. Hypertrophic chondrocytes are able to survive the transition of cartilage into bone and become osteoblasts and osteocytes during endochondral bone formation [74].

Our bioinformatics analysis revealed that SOX-9 is at the center of a complex regulatory network and interacted with few proteins involved in the development of OA. One of the proteins is *secreted phosphoprotein 1 (SPP-1)*, *also called* osteopontin (OPN). Luciferase assays performed by Peacock et al. showed that SOX-9 represses the promoter activity of *SPP1* [75]. This glycoprotein participates in the development and maintenance of metabolic homeostasis and the repair of articular cartilage [76]. Also, it was revealed SPP-1 regulates the levels of hyaluronic acid (HA), type II collagen, proteoglycans, interleukin-1 (IL-1), nitric oxide (NO), prostaglandin E2 (PGE2), hypoxia-inducible factor-2*α* (HIF-2α) and acts as a disintegrin and metalloproteinase with thrombospondin motifs 4 (ADAMTS-4) and as a protective factor in the development of OA [77,78,79,80]. Moreover, Liu et al. reported that SPP-1 activates the intracellular PI3K signaling pathway by binding to CD44 on the cell surface and inhibiting OA progression via delaying the degeneration of chondrocyte and decreasing the loss of cartilage matrix components [81]. However, SPP-1 has also been found to promote calcium pyrophosphate dihydrate deposition in cartilage tissue and enhance the production of matrix metallopeptidase 13 (MMP-13) and the activation of the nuclear factor-kappa B (NF-κB) pathway, causing intensify OA progression [82,83]. SPP1 mRNA and protein expression levels were also elevated in plasma, synovial fluid, and articular cartilage in OA patients [84,85,86]. Therefore, the exact role of SPP1 in the development and progression of OA remain unclear.

Furthermore, our analysis also found an interaction between SOX-9 with cyclin D1. Previous studies have shown that SOX-9 also plays a key role in controlling proliferation through transcriptional regulation of *CCND1*, coding cyclin D1 [87]. SOX-9 competes with T-cell factor/lymphoid enhancer factor (*TCF*/*LEF*) transcription factors for binding to beta-catenin. Blocking beta-catenin by SOX-9 prevents the formation of the beta-catenin/TCF complex that binds to the TCF/LEF consensus site in the cyclin D1 promoter, transactivating *CCND1*. Cyclin D1 is an important regulator of the cell cycle, enabling the induction of cell entry into the S phase and G2/M phase of the cell cycle.

Our bioinformatics data indicate a relationship between SOX-9 and the lysyl oxidase family (LOX); these are copper-dependent enzymes which play essential roles in cartilage homeostasis by regulating ECM remodeling and collagen cross-linking [88]. Alshenibr et al. reported that appropriate LOX mRNA and expression levels are extremely important for the functional integrity of articular cartilage [89]. The increase in the expression level of *LOXL2* mRNA in OA-affected chondrocytes resulted in a higher expression level of *SOX9* mRNA and other genes such as *CSPG4* (coding chondroitin sulfate proteoglycan) and *ACAN*, which may be a protective response that promotes anabolism response in the pathophysiology of OA.

In our analysis, we also found a common interaction network between SOX-9, LOX, and IGF-II in chondrocytes. A recent study by Waldrep et al. showed that insulin-like growth factor II (IGF-II) regulates the expression of LOX enzymes, which increase the expression of various ECM components, such as COL3A1, as well as collagen post-transcriptional modification enzymes, including propyl-4-hydroxylase alpha 2 subunit (P4HA2) in a SOX9-dependent manner [90]. *SOX9* is an immediate early gene activated by IGF-II through the IGF-1R/IR hybrid receptor. Uchimura et al. found IGF-II to have a protective role by inhibiting IL-1β-induced cartilage matrix loss and promoting cartilage integrity [91].

Our bioinformatics analysis also associated SOX-9 with integrin β1 (ITGβ1), which has been confirmed previously. Luo et al. reported that *SOX9* mRNA expression is also regulated by ITGβ1 [92]. Increased *ITGB1* mRNA expression level in adipose tissue-derived mesenchymal stem cells (ADSCs) transfected with recombinant plasmids (LV003-ITGB1) enhanced the expression of SOX-9 at both the mRNA and protein level. Furthermore, their findings indicate that ITGβ1 plays a key role in regulating the chondrogenic differentiation of ADSCs via activation of the extracellular signal-regulated kinase (ERK) signaling pathway. In vitro and in vivo studies of Xie et al. revealed that ITGβ1 alleviates OA by activating the cyclic adenosine 3′,5′-monophosphate (cAMP) signaling pathway, thus inhibiting cartilage inflammation and apoptosis. Local intra-articular injection of cells transfected with a lentivirus overexpressing *ITGB1* decreased inflammatory injury and inhibited OA progression in Sprague Dawley male rats; this suggests that ITGβ1 promotion may be an effective therapy target of OA in the future [93].

In addition, our data indicated that SOX-9 interacts with the FOS and JUN families of DNA-binding proteins, which are part of the activator protein-1 (AP-1) transcription factor complex. Other studies by Hwang et al. found that c-JUN/AP-1 modulated the *COL2A1* mRNA expression level by regulating *SOX9* mRNA expression levels [94]. c-JUN expression and phosphorylation suppressed SOX-9 expression at the mRNA and protein levels, thus reducing the promoter activity of *COL2A1*, which may ultimately lead to cartilage destruction. However, He et al. showed that AP-1 family members demonstrate a degree of similarity with the SOX9-bound region and together are engaged in the hypertrophic transition of chondrocytes [95]. Their in vitro study showed the direct co-binding of SOX-9 and AP-1 at target motifs, which promoted the gene activity of *COL10A1*.

So far, several studies have been conducted to evaluate differences in SOX family member gene expression levels between normal and osteoarthritic cartilage from adult humans [26,41,42,71]. A study by Haag et al. aimed to assess the expression level of 19 members of the SOX TF family genes and found most of them to demonstrate very weak expression, or none at all, in both in unaffected cartilage and cartilage affected by OA [41]. The highest relative expression level of *SOX9* mRNA compared to *GAPDH* mRNA, as an endogenous control, was noted in normal cartilage; however, *SOX9* mRNA demonstrated lower expression in OA-affected cartilage compared to normal tissue. Quyang et al. also reported lower *SOX9* mRNA expression level in OA-affected cartilage compared to unaffected tissue [96]. In turn, Zhang et al. demonstrated that *SOX9* mRNA expression level significantly varied according to the progression of OA [71]. Their results demonstrated a significant increase in *SOX9* mRNA expression level in articular cartilage at stage 1 of disease (assessed according to Outerbridge classification) compared to unaffected cartilage; subsequently, its expression level decreased as the disease progressed from stage 2 to stage 4. In the present study, *SOX9* mRNA expression level was found to be significantly elevated in OA-affected cartilage with subchondral bone compared to control tissue in patients with advanced stages of disease. Additionally, a higher expression level of *SOX9* mRNA was noted in OA-affected cartilage with subchondral bone of patients with stage 4 of the disease compared to those with stage 3 (assessed according to Kellgren–Lawrence classification). The Kellgren–Lawrence classification is currently the most widely used clinical tool for the radiographic diagnosis of OA, and it appears to most closely reflect the state of the disease in the medial compartment of the knee compared to Outerbridge scores [97]. However, further studies using the Kellgren–Lawrence classification are needed to confirm our results.

The study by Haag et al. also found moderate expression levels of *SOX5* mRNA in normal articular cartilage, but the expression level of this gene in cartilage from OA patients was elevated [41]. Similarly, our present findings indicate a significantly higher expression level of *SOX5* mRNA in OA-affected cartilage with subchondral bone compared to control tissue. Furthermore, the *SOX5* expression level increased with OA progression, but the differences observed between stage 4 and stage 3 (according to Kellgren–Lawrence classification) were statistically insignificant. Although some previous studies have reported significantly increased expression level of *SOX5* mRNA in OA cartilage tissue compared to normal tissue, none assessed the changes according to OA progression [98,99,100]. SOX-5 expression was found to be negatively regulated by various miRNAs, *viz.* miR-193a-3p, miR-142-5p, and miR-197-3p; however, their expression in chondrocytes decreased as OA progressed. The decrease in the expression level of these miRNA contributed to an elevated expression *SOX5* mRNA and thus decreased cell proliferation and enhanced apoptosis, ECM degradation, and inflammation by increasing the expression of pro-inflammatory cytokines, such as tumor necrosis factor alpha (TNF-α) and interleukin-6 (IL-6), which may promote OA progression.

Haag et al. reported the expression level of *SOX11* mRNA to be below detection in normal cartilage but present in OA-affected articular cartilage [41]. Our present findings show a higher *SOX11* mRNA expression level in OA-affected cartilage with subchondral bone compared to control cartilage. The differences in *SOX11* mRNA expression level in cartilage samples were statistically insignificant, which is consistent with the results of Fu et al. [101], but Xu et al. noted significantly higher *SOX11* mRNA expression in OA cartilage compared to normal cartilage [26]. Moreover, our findings revealed the increase in *SOX11* mRNA expression in OA-affected cartilage with subchondral bone with disease progression. An in vitro study by Xu et al. on a human chondrocyte cell line (CHON-001 cells) demonstrated that *SOX11* mRNA expression level increased after stimulation with IL-1β, a pro-inflammatory cytokine, which was associated with induction of chondrocytes apoptosis. Inhibition of *SOX11* expression with appropriate siRNAs alleviated IL-1β-induced apoptosis of CHON-001 cells, decreased both the protein and mRNA expression level of MMP-13, cleaved caspase 3, and increased COL2A1 and ACAN expression levels via regulation of the TNF-α expression. The above results suggest that TNF-α is a downstream effector of SOX-11 and that it may have a role in promoting OA progression.

Obesity remains one of the most important preventable risk factors for the occurrence and progression of OA [102,103] and has been found to double the lifetime risk of developing symptomatic OA compared to people with a BMI below 25 [104]. It is believed to increase the risk of OA by overloading the joints, in turn leading to the destruction of joint cartilage. However, recent studies have shown that various other factors, such as fat deposition, insulin resistance, and inflammatory components caused by the improper coordination of innate and adaptive immune responses, can lead to the initiation and progression of obesity-related OA [103]. Adipose tissue (AT) secretes adipokines, including adiponectin, which has recently attracted much scientific interest due to its involvement in the regulation of chondrocyte growth, as well as in their differentiation and production of ECM-degrading enzymes and other cytokines that may ultimately lead to the development of OA [105]. Higher adiponectin expression was found in serum from patients with OA compared to controls, and its levels correlated positively with disease progression (according to the Kellgren–Lawrence grading system) [106,107,108]. In vitro studies found adiponectin increases the expression level of *SOX9* in human chondrocytes and may actively participate in degradation of the cartilage matrix and the chondrocyte hypertrophic transformation in OA [109].

Our study revealed only *SOX5* mRNA expression level in OA-affected articular cartilage with subchondral bone was positively correlated with BMI; however, we noticed *SOX9* and *SOX11* mRNA expression level was higher in patients with class II + III obesity compared to patients with class I obesity. These are the first findings regarding changes in the expression level of the studied SOX family TFs with regard to body weight and the degree of obesity in OA patients (according to BMI). Adiponectin may increase not only the *SOX9* mRNA expression level, but also other genes coding SOX family TFs; however, this hypothesis requires further study.

Aging is accompanied by many functional declines at the molecular, cellular, tissue, and organismal levels. One important epigenetic phenomenon is aging-associated DNA methylation changes found at TF-binding sites; this is commonly observed in the HMG domains of SOX family TFs [110,111]. It has been recently shown that the expression of some TF members belonging to the *SOX* family change with age in various cells and tissues [111,112,113]. *SOX2* expression level has been found to decrease in different tissues in humans, including the brain and peripheral blood mononuclear cells (PBMCs) during aging; in addition, *SOX2* expression level may be a biological marker of aging [113]. Moreover, studies on luminal epithelial cells in mammary tissue have demonstrated a decrease in *SOX4* expression level with aging [111]. In the present study, all studied genes (*SOX5*, *SOX9*, and *SOX11*) demonstrated lower expression at the mRNA level in OA-affected cartilage with subchondral among OA patients aged over 65 years compared to those aged 65 years or below. The differences between the groups were statistically insignificant.

The adult synovium is a site where the synovium houses mesenchymal stem cells (SM MSC), highly clonogenic cells with chondrogenic potential, expressing SOX-5, SOX-6, SOX-9, which can move to the site of injured cartilage and promote its repair [114,115]. The OA-affected joint tends to exhibit an increased number of native resident MSCs in the synovium and synovial fluid, but the expression level of the SOX TF family at the mRNA level in these cells is reduced, leading to an inhibition of their proliferation rate, a reduction in their self-renewal capacity and cartilage differentiation potential [114,116,117,118], and an inhabitation of their ability to repair degraded cartilage [115,116]. Our present findings indicate a significant reduction in *SOX5* and *SOX11* mRNA expression levels in OA-affected synovium compared to the control tissue; this was accompanied by a reduction in *SOX9* mRNA expression level; however, this difference was not significant, and *SOX9* mRNA expression decreased with OA progression. Increasing the expression level of the tested genes in synovial articular progenitor cells may be a therapeutic goal for patients with OA.

The efficiency of MSCs falls with age and eventually becomes insufficient to support tissue or organ regeneration [119]. In seniors, MSCs also present different properties to those from younger donors, and their numbers decline with age [119,120]. They also demonstrate lower colony-forming efficiency, proliferative capacity, and chondrogenic differentiation potentials [121]. Our present findings also indicate reduced expression of all studied genes (*SOX5, SOX9*, and *SOX11*) in OA-affected synovium according to aging; however, the differences in their expression between the study group of patients were statistically insignificant.

There is increasing evidence that inflammation in the *synovium* plays a major role in the progression of *OA*. Physiologically, fibroblast-like synoviocytes (FLSs) specialize in the production of lubricin, hyaluronan, and heparan sulfate proteoglycans; however, during the development of OA, they transform into an aggressive phenotype and produce inflammatory cytokines and catabolic enzymes, enhance proliferation, and avoid apoptosis and the host immune system [122]. Some SOX family members of TFs, such as SOX-5, SOX-4, and SOX-11, are believed to play a role in FLS transformation [123,124]. In the presence of pro-inflammatory factors such as IL-6, the increase in the *SOX5* mRNA expression level promotes the migration and invasive properties of human FLSs by regulating *RANKL* and *MMP9* mRNA expression levels, which may cause ECM invasion, leading to joint destruction in patients with rheumatoid arthritis (RA) [123,124]. However, no studies have examined the change in *SOX5* mRNA expression level occurring in the synovium during the course of OA. Our present findings indicate that *SOX5* mRNA expression level in the synovium increases as OA progresses from stage 3 to stage 4, according to the Kellgren–Lawrence classification. Similarly, a higher *SOX11* mRNA expression level was noted in the synovium of OA patients with more advanced stages of disease. Bhattaram et al. reported that the increased expression of SOX-4 and SOX-11 in FLSs amplify the pathogenic impact of TNF by increasing their survival and migration [122]. Our results suggest that increased expression levels of *SOX5* and *SOX11* mRNA in the synovium may indicate advanced stages of OA. The increase in expression levels of these genes may be associated with FLS transformation.

Obesity is an important factor that promotes the acquisition inflammatory phenotype of FSLs [125,126]. The increase in level of pro-inflammatory cytokines, including IL-6, which are observed in the synovial joint fluid (SF) of obese OA patients, may lead to the increase in expression levels of SOX family TFs genes in FLSs [117]. In our study, a positive correlation was observed between *SOX5* and *SOX9* mRNA expression level and BMI. In addition, higher *SOX11* mRNA expression levels were noted in the synovium of OA patients with class I obesity compared those who were overweight; however, its expression level was lower than that noted in patients with class II +III obesity. These findings suggest that the change in *SOX5* and *SOX9* mRNA expression level in the synovium of patients with OA may also result from the influence of obesity-related factors.

It should be noted that relatively few statistically significant results were obtained regarding the relationship between the expression of the studied genes and the clinical characteristics of OA patients. This may have been due to the relatively small number of patients involved in the study (n = 31). The observed trends in changes in the expression of the studied genes should be confirmed in further studies conducted on larger groups of patients. Furthermore, due to the difficulties in separating the very thin and damaged cartilage tissue from the adjacent subchondral bone during surgery, these tissues were analyzed as a complex.

## 5. Conclusions

Our bioinformatics analysis show a regulatory network between SOX-5, SOX-9, and SOX-11 and various proteins engaged in the pathogenesis of knee OA as well as their collective interactions, which are involved in the regulation of cartilage extracellular matrix (ECM) organization, response to stimulus, regulation of gene expression, inflammatory response, cartilage condensation, and ossification in chondrocytes. The significant changes in the studied gene expression level in OA-affected articular cartilage with subchondral bone and synovium indicate the development of pathological changes in the entire joint. The increase in the expression level of *SOX5*, *SOX9*, and *SOX11* in articular cartilage with subchondral bone in a group of patients with a more advanced stage of disease may be related to enhanced the transition of chondrocytes into hypertrophic phenotype and their apoptosis and may contribute to endochondral bone formation and degradation of articular cartilage. Decreased expression levels of the studied genes’ mRNA in synovium may be related to reductions in the chondrogenic capacity of MSCs during the development of OA; however, the increase in *SOX5* and *SOX11* mRNA expression level with the progression of disease may be associated with enhanced FLS transformation. The assessment of the *SOX5*, *SOX9*, and *SOX11* mRNA expression level in articular cartilage with subchondral bone and synovium may be used as potential molecular diagnostic biomarkers for detecting OA in the future; however, our observations require further investigation on a larger group of patients.

## Figures and Tables

**Table 1:** Inclusion and exclusion criteria for the study.

Inclusion Criteria
Diagnosis of primary knee OA with predominantly medial involvement
Advanced degenerative changes found in the radiological examination The third or fourth degree of disease, according to Kellgren–Lawrence classification
Giving voluntary, informed, written consent to participate in the study
**Exclusion criteria**
Rheumatoid arthritis
Hemophilia
Psoriatic arthritis
Neurological disorders

**Table 2:** The clinical characteristics of the patients with diagnosed primary knee OA.

Patient Characteristics	Number of Patients (n)	Total Percentage of Patients
Sex		
Female	21	68%
Male	10	32%
Age		
≤65 years	14	45%
>65 years	17	55%
Weight		
≤88 kg	16	52%
>88 kg	15	48%
BMI ^a^		
25.0–29.9 (overweight)	4	13%
30.0–34.9 (class I obesity)	20	65%
35.0–39.0 (class II obesity)	6	19%
≥40 (class III obesity)	1	3%
Stage of knee OA according to Kellgren–Lawrence classification		
Stage 3	14	45%
Stage 4	17	55%

^a^ BMI, calculated by dividing the body weight in kilograms by height in meters squared [29].

**Table 3:** Median expression level (RQ value) of the studied genes in OA-affected articular cartilage with subchondral bone vs. control tissue with regard to the clinical and pathological characteristics of patients.

	*SOX5*		*SOX9*		*SOX11*	
	(Median RQ)		(Median RQ)		(Median RQ)	
OA-affected articular cartilage with subchondral bone control tissue		*p* = 0.00015		*p* = 0.0024		*p* > 0.05
	237.935		522.929		354.66	
		
	69.395		178.271		317.992	
female	220.128	*p* > 0.05	556.385	*p* > 0.05	354.66	*p* > 0.05
male	339.09		400.887		332.913	
≤65 years	339.09	*p* = 0.04	606.861	*p* = 0.017	372.414	*p* > 0.05
>65 years	189.304		272.911		285.509	
Overweight	210.926	*p* > 0.05	478.408	*p* > 0.05	556.954	*p* > 0.05
Class I obesity	285.76		459.286		280.584	
Class II + III obesity	310.183		800.773		390.167	
Stage 3	240.662	*p* > 0.05	348.422	*p* > 0.05	280.584	*p* > 0.05
Stage 4	287.935		585.338		390.167	

**Table 4:** Median expression level (RQ value) of studied genes in OA-affected synovium vs. control tissue and according to the clinical characteristics of patients.

	*SOX5*		*SOX9*		*SOX11*	
	(Median RQ)		(Median RQ)		(Median RQ)	
OA-affected synovium	10.193	*p* = 0.0003	19.722	*p* > 0.05	12.436	*p* = 0.0007
Control tissue	23.297		22.874		44.471	
female	11.056	*p* > 0.05	24.511	*p* > 0.05	10.717	*p* > 0.05
male	7.354		14.373		15.003	
≤65 years	12.583	*p* > 0.05	20.699	*p* > 0.05	15.003	*p* > 0.05
>65 years	8.271		19.699		5.8	
Overweight	4.265	*p* > 0.05	14.333	*p* > 0.05	6.308	*p* > 0.05
Class I obesity	9.281		21.511		12.875	
Class II + III obesity	14.658		21.849		6.95	
Stage 3	5.884	*p* > 0.05	20.786	*p* > 0.05	11.577	*p* > 0.05
Stage 4	11.674		19.549		12.746	

## Data Availability

The data used to support the findings of this study are available from the corresponding author upon request.

## Supplementary Materials

The following supporting information can be downloaded at: https://www.mdpi.com/article/10.3390/jcm14041176/s1, Figure S1: Identification of OA-associated up- and down-regulated genes in chondrocytes and synoviocytes.
