# Supplementary material for: The Expression Level of SOX Family Transcription Factors’ mRNA as a Diagnostic Marker for Osteoarthritis
Source: J Clin Med. 2025 Feb 11;14(4):1176. doi: 10.3390/jcm14041176 (PMC11856735; doi:10.3390/jcm14041176)
Supplement: Supplementary file 1 [file jcm-14-01176-s001.zip › jcm-3411700-supplementary.pdf]

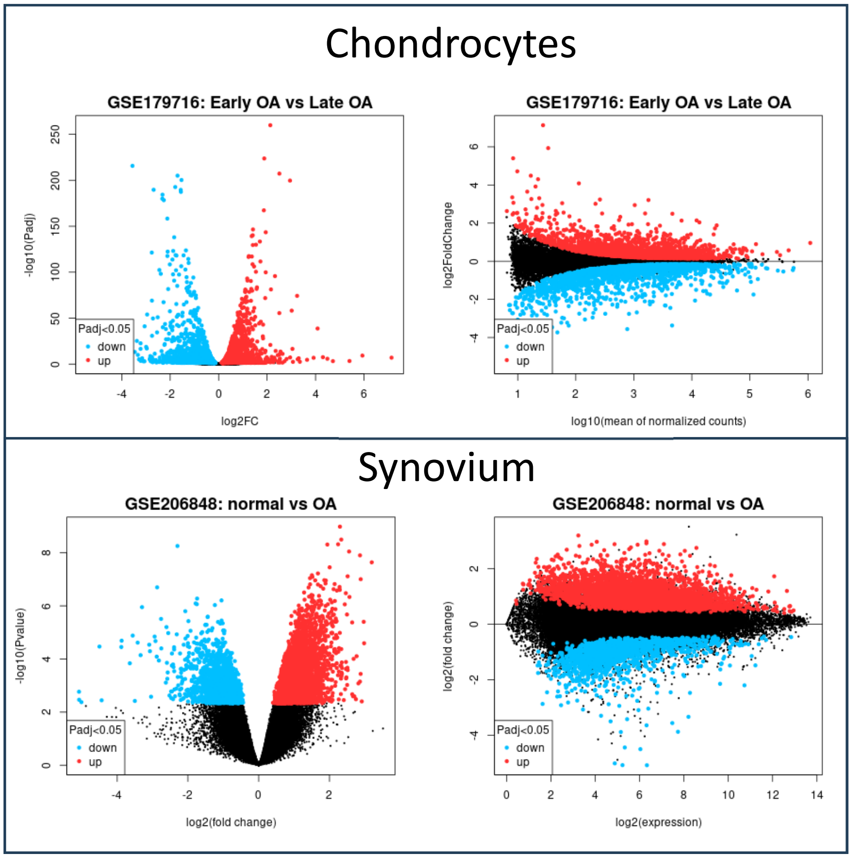

Figure S1. Identification of OA-associated up- and down-regulated genes in chondrocytes and synoviocytes.

Top up- and down-regulated genes from OA-affected chondrocytes and synoviocytes were identified using GEO2R online analytical tool and presented as volcano plots for each data set.
